# *CLPTM1L* gene rs402710 (C > T) and rs401681 (C > T) polymorphisms associate with decreased cancer risk: a meta-analysis

**DOI:** 10.18632/oncotarget.22268

**Published:** 2017-11-01

**Authors:** Jianzhou Tang, Changming Hu, Hua Mei, Liang Peng, Hui Li

**Affiliations:** ^1^ Department of Biological and Environmental Engineering, Changsha University, Changsha 410003, Hunan, China; ^2^ Department of Molecular Pathology, Guangzhou Kingmed Center for Clinical Laboratory, Guangzhou 510000, Guangdong, China; ^3^ Department of Somatic Stem Cell, Hunan Guangxiu Hospital, Changsha 410002, Hunan, China; ^4^ Department of Microbiology and Immunology, Medical School of Jishou University, Jishou 416000, Hunan, China

**Keywords:** CLPTM1L, polymorphism, cancer, risk, meta-analysis

## Abstract

*Cleft lip and palate transmembrane 1-like* (*CLPTM1L*) gene rs402710 (C > T) and rs401681 (C > T) polymorphisms have been widely studied for their potential relation to cancer risk, but studies have produced conflicting results. To systematically evaluate the association between these two polymorphisms and overall cancer risk, we conducted a comprehensive meta-analysis on all relevant articles found in the PubMed and EMBASE databases published prior to May 1, 2017. There were 26 articles with 28 studies, including 30,770 cases and 34,089 controls, for the rs402710 polymorphism and 38 articles with 48 studies, including 67,849 cases and 328,226 controls, for the rs401681 polymorphism. The pooled results indicated that both rs402710 and rs401681 polymorphisms are significantly associated with decreased overall cancer risk. In our stratification analysis, a significant association of the rs402710 polymorphism with lung and bladder cancers was identified among Asian and Caucasian populations in both hospital-based and population-based studies. The rs401681 polymorphism was significantly associated with a decreased risk of lung cancer, bladder cancer, and basal cell carcinoma in Asians and in hospital-based studies. *CLPTM1L* gene rs402710 and rs401681 polymorphisms thus have a protective association with various types of cancer, especially lung cancer among Asians.

## INTRODUCTION

The number of people who will develop and die from cancer is expected to climb rapidly around the world [1]. In 2016, a total of 1,685,210 cases of cancer were newly diagnosed and 595,690 people died from cancer in the United States [2]. Meanwhile, approximately 4,292,000 new cancer cases and 2,814.000 cancer deaths were projected in China in 2015 [3]. It is imperative to deal with the rising cancer burden by taking full advantage of the knowledge embedded in cancer statistics, causes, and mechanisms and apply it to cancer prevention and screening [4]. Genetic-environmental interaction has been considered an essential factor in carcinogenesis [5]. Meanwhile, the roles of multiple genetic changes in influencing the evolution of cancer have been investigated over the years [6].

Recently, a number of investigations have been conducted to study the potential influence of *Telomerase reverse transcriptase* (*TERT*) and *cleft lip and palate transmembrane 1-like* (*CLPTM1L*) gene variation on cancer susceptibility. The *TERT* gene encodes the rate-limiting catalytic subunit of the telomerase enzyme that is vital to the maintenance of telomere DNA length, chromosomal stability, and cellular immortality [7]. *CLPTM1L*, encodes a protein linked to cisplatin resistance and associated with the susceptibility to cleft lip palate. *CLPTM1L* was found to be overexpressed in cisplatin-resistant ovarian cancer cells and to promote apoptosis in cisplatin-sensitive cells [8]. *TERT-CLPTM1L* genetic variants, including single nucleotide polymorphisms, have been reported by several genome-wide association studies (GWAS) to associate with the risk of multiple cancer types [9–11]. Among these polymorphisms, rs402710 (C > T) and rs401681 (C > T) have been widely studied for their potential effect on the susceptibility to cancer. Jiang et al. found that rs401681 polymorphism was associated with a decreased risk of lung cancer [12], but other studies found no association was found between rs401681 polymorphism and lung cancer risk [13, 14]. For the rs402710 polymorphism, Ito et al. observed remarkable effects on susceptibility in lung cancer [15], but the results were not repeated in other similar studies [16, 17]. We conducted a comprehensive meta-analysis of all relevant articles to provide a systematic and cumulative assessment of the association of *CLPTM1L* rs402710 and rs401681 polymorphisms and overall cancer risk.

## RESULTS

### Study characteristics

**Figure 1:**
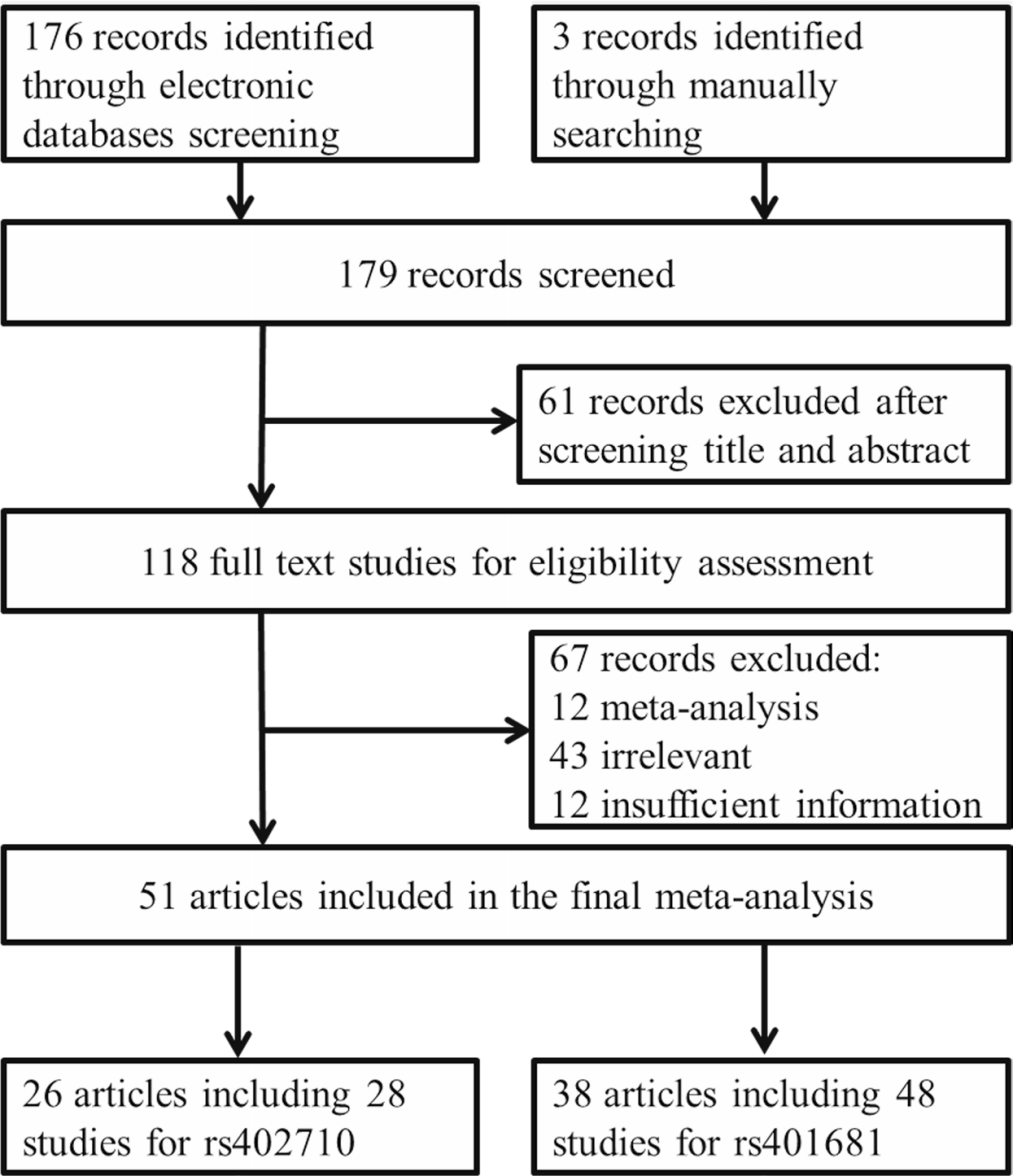
Flow diagram of studies included in our meta-analysis

As shown in Figure 1, 176 publications were initially identified from PubMed and EMBASE electronic databases that met our inclusion and exclusion criteria. After title and abstract screening, 61 records were excluded. The remaining 115 full text articles were further assessed and 67 were further excluded for the following reasons: 1) 12 were meta-analyses; 2) 43 were irrelevant; and 3) 12 had insufficient information to calculate odds ratios (ORs) and 95% confidence intervals (95% CIs). Additionally, 3 relevant studies were also retrieved manually from the pool. In the end, 51 eligible articles were included in the final meta-analysis.

The genotypic distribution of rs402710 and rs401681 polymorphisms in the controls in almost all of the studies followed Hardy-Weinberg equilibrium (HWE); four did not (Table 1) [11, 17–19]. Since the genotypic distribution of other polymorphisms was in agreement with HWE in these four studies, the investigators decided to include them in the final analysis. Together, there were 26 articles with 28 studies including 30,770 cases and 34,089 controls for the rs402710 polymorphism [13, 15–39] and 38 articles with 48 studies including 67, 849 cases and 328, 226 controls for the rs401681 polymorphism (Table 1) [9–12, 14, 16–18, 21, 23–25, 28, 29, 32, 34, 35, 37, 38, 40–58]. For the rs402710 polymorphism, 19 studies focused on lung cancer, 2 on bladder cancer, and the rest on other individual types of cancer. There were 21 studies conducted on Asian populations, 5 on Caucasians, and 2 on Africans. 15 were hospital-based, and 12 were population-based. One was nested. As to the rs401681 polymorphism, 20 studies focused on lung cancer, 5 on bladder cancer and pancreatic cancer, 4 on melanoma, 3 on basal cell carcinoma, 2 on esophageal cancer and squamous cell carcinoma, and 7 on other individual cancer types, which we grouped as “the others” for our analyses. 24 were conducted on Asians, 21 on Caucasians, 2 on Africans, and only one on mixed ethnicity. There were 19 studies with a hospital-based design, 21 with a population-based design, and 8 with a nested design.

**Table 1:** Characteristics of case-control studies included in the current meta-analysis

Surname	Year	Country	Ethnicity	Cancer type	Control source	Genotyping method	Cases/Controls		HWE
**rs402710 polymorphism**									
Zhou	2016	China	Asian	ESCC	HB	MassArray	588	600	0.762
Zhang	2016	China	Asian	NPC	HB	MassArray	855	1036	0.512
Jin	2016	China	Asian	Lung cancer	HB	MassArray	552	717	0.007
Ge	2016	China	Asian	Thyroid carcinoma	HB	MassArray	500	500	0.675
Liu	2015	China	Asian	Lung cancer	PB	MassArray	288	318	0.997
Zhao	2014	China	Asian	Lung cancer	HB	Taqman	951	954	0.502
Xun	2014	China	Asian	Lung cancer	HB	MassArray	227	300	0.795
Liang	2014	China	Asian	Lung cancer	HB	MassArray	308	309	0.670
Zhao	2013	China	Asian	Lung cancer	PB	SNPscan	784	782	/
Lu	2013	China	Asian	Lung cancer	HB	Taqman	604	1062	0.971
Zheng	2012	USA	African	Breast cancer	PB	Illumina	1509	1383	/
Zhao	2012	China	Asian	Glioma	HB	MassArray	452	486	0.308
Wang	2012	China	Asian	Cervical cancer	HB	Taqman	1022	1046	0.741
Ren	2012	China	Asian	Prostate cancer	HB	PCR-RFLP	251	273	0.210
Ito	2012	Japan	Asian	Lung cancer	HB	Taqman	716	716	0.712
Chen	2012	China	Asian	Lung cancer	PB	Taqman	193	228	0.016
Bae	2012	Korea	Asian	Lung cancer	PB	Melting curve	1094	1099	0.682
Pande	2011	USA	Caucasian	Lung cancer	HB	Illumina	1681	1235	/
Jaworowska	2011	Poland	Caucasian	Lung cancer	HB	Taqman	848	845	0.696
Gago-Dominguez 1	2011	USA	African	Bladder cancer	PB	Taqman	471	528	0.109
Gago-Dominguez 2	2011	USA	Asian	Bladder cancer	PB	Taqman	503	527	0.223
Yoon	2010	Korea	Asian	Lung cancer	PB	Taqman	1425	3011	0.356
Truong 1	2010	France	Caucasian	Lung cancer	PB	Taqman	8860	9198	0.961
Truong 2	2010	France	Asian	Lung cancer	PB	Taqman	1680	2117	0.828
Liu	2010	USA	Caucasian	Lung cancer	PB	Affymetrix	194	214	/
Hsiung	2010	China	Asian	Lung cancer	Nested	Illumina	2659	2844	0.001
Zienolddiny	2009	Norway	Caucasian	Lung cancer	PB	Taqman	356	432	0.787
Jin	2009	China	Asian	Lung cancer	HB	PCR-RFLP	1199	1329	0.578
**rs401681 polymorphism**									
Zhang	2016	China	Asian	NPC	HB	MassArray	1852	2008	0.832
Jin	2016	China	Asian	Lung cancer	HB	MassArray	554	695	0.411
Liu	2015	China	Asian	Lung cancer	PB	MassArray	292	319	0.696
Gibbs	2015	USA	Caucasian	Melanoma	PB	MassArray	1187	2409	0.512
Campa	2015	Germany	Caucasian	Pancreatic cancer	PB	Illumina	1857	4048	0.482
Zhang	2014	China	Asian	Lung cancer	HB	Taqman	366	364	0.097
Zhang	2014	China	Asian	Bladder cancer	HB	PCR–LDR	367	420	0.683
Yin	2014	China	Asian	Esophageal cancer	HB	PCR–LDR	604	664	0.365
Xun	2014	China	Asian	Lung cancer	HB	MassArray	224	299	0.819
Su	2014	China	Asian	HCC	HB	Taqman	201	210	0.337
Llorca-Cardenosa	2014	Spain	Caucasian	Melanoma	HB	Taqman	956	722	0.263
Liu	2014	China	Asian	Pancreatic cancer	PB	Taqman	766	821	0.263
Liang	2014	China	Asian	Lung cancer	HB	MassArray	309	308	0.631
Wang	2013	China	Asian	Lung cancer	HB	HRM	492	486	0.076
Sun	2013	China	Asian	Lung cancer	HB	MassArray	400	200	0.942
Myneni	2013	China	Asian	Lung cancer	HB	MassArray	350	441	0.668
Ma	2013	China	Asian	Bladder cancer	HB	MassArray	177	920	0.044
Li	2013	China	Asian	Lung cancer	HB	Taqman	464	536	0.865
Ke	2013	China	Asian	Lung cancer	PB	Taqman	602	1060	0.939
Jiang 1	2013	China	Asian	Lung cancer	HB	Taqman	726	860	0.805
Jiang 2	2013	China	Asian	Esophageal cancer	HB	Taqman	753	860	0.805
Zheng	2012	USA	African	Breast cancer	PB	Illumina	1509	1383	/
Zhao	2012	China	Asian	Glioma	HB	MassArray	983	1024	/
Lan	2012	USA	Asian	Lung cancer	Nested	Illumina	5382	4452	/
Chen	2012	China	Asian	Lung cancer	PB	Taqman	195	228	0.016
Bae	2012	Korea	Asian	Lung cancer	PB	Melting curve	1086	1079	0.162
Willis	2012	USA	Caucasian	Pancreatic cancer	HB	Illumina	390	149	/
Rizzato	2011	Germany	Caucasian	Pancreatic cancer	PB	AS-PCR	661	1267	0.949
Pande	2011	USA	Caucasian	Lung cancer	HB	Illumina	1681	1235	/
Nan 1	2011	USA	Caucasian	Melanoma	PB	Taqman	208	809	0.497
Nan 2	2011	USA	Caucasian	SCC	PB	Taqman	266	809	0.497
Nan 3	2011	USA	Caucasian	BCC	PB	Taqman	283	809	0.497
Gago-Dominguez 1	2011	USA	African	Bladder cancer	PB	Taqman	472	554	0.686
Gago-Dominguez 2	2011	USA	Asian	Bladder cancer	PB	Taqman	500	529	0.292
Yoon	2010	Korea	Asian	Lung cancer	PB	Taqman	1425	3011	0.217
Petersen	2010	USA	Mixed	Pancreatic cancer	Nested	Illumina	3532	3642	/
Liu	2010	USA	Caucasian	SCCHN	PB	Taqman	1079	1115	0.775
Liu	2010	USA	Caucasian	Lung cancer	PB	Affymetrix	194	214	/
Zienolddiny	2009	Norway	Caucasian	Lung cancer	PB	Taqman	341	431	0.366
Stacey 1	2009	Iceland	Caucasian	Melanoma	Nested	Illumina	3843	41963	/
Stacey 2	2009	Iceland	Caucasian	SCC	PB	Illumina	1103	35824	/
Stacey 3	2009	Iceland	Caucasian	BCC	Nested	Illumina	3468	38107	/
Rafnar 1	2009	Iceland	Caucasian	BCC	Nested	Illumina	2565	29405	/
Rafnar 2	2009	Iceland	Caucasian	Lung cancer	Nested	Illumina	4265	34666	/
Rafnar 3	2009	Iceland	Caucasian	Bladder cancer	Nested	Illumina	4147	34988	/
Rafnar 4	2009	Iceland	Caucasian	Prostate cancer	Nested	Illumina	9473	37901	/
Rafnar 5	2009	Iceland	Caucasian	Cervical cancer	PB	Illumina	276	28890	/
Wang	2008	UK	Caucasian	Lung cancer	PB	Illumina	5023	5092	0.223

ESCC, esophageal squamous cell carcinoma; NPC, nasopharyngeal carcinoma; HCC, hepatocellular carcinoma; SCC, squamous cell carcinoma; BCC, basal cell carcinoma; SCCHN, squamous cell carcinoma of the head and neck; HB, hospital based; PB, population based; PCR-RFLP, polymerase chain reaction-restriction fragment length polymorphism; PCR–LDR, polymerase chain reaction–ligation detection reaction; HRM, high-resolution melt; AS-PCR, allele-specific polymerase chain reaction; HWE, Hardy-Weinberg equilibrium.

### Meta-analysis results of the rs402710 polymorphism

**Figure 2:**
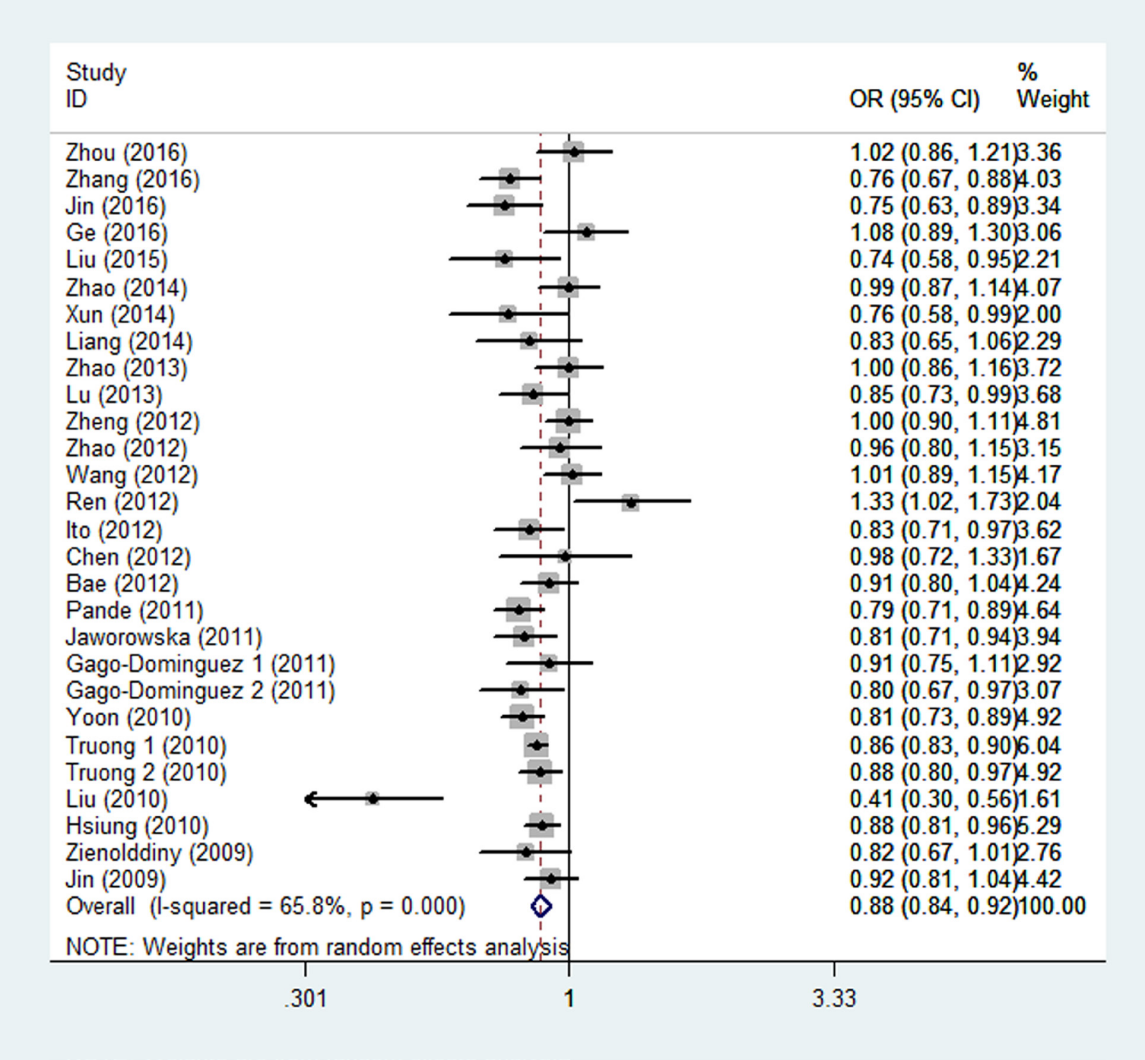
Forest plot of the association between *CLPTM1L* rs402710 polymorphism and overall cancer risk under the allele contrast model

As shown in Table 2 and Figure 2, there was evidence of a significant association between the rs402710 polymorphism and overall cancer risk under all of the five genetic models [homozygous (TT vs. CC): OR = 0.77, 95% CI = 0.70–0.84; heterozygous (CT vs. CC): OR = 0.89, 95% CI = 0.86–0.92; recessive (TT vs. CT+CC): OR = 0.81, 95% CI = 0.74–0.88; and dominant (CT+TT vs. CC): OR = 0.87, 95% CI = 0.83–0.91; as well as the allele comparison model (T vs. C): OR = 0.88, 95% CI = 0.84–0.92]. In the stratification analysis by cancer type, a statistically significant association was identified with lung cancer [homozygous (TT vs. CC): OR = 0.73, 95% CI = 0.67–0.79; heterozygous (CT vs. CC): OR = 0.88, 95% CI = 0.85–0.91; recessive (TT vs. CT+CC): OR = 0.77, 95% CI = 0.71–0.84; and dominant (CT+TT vs. CC): OR = 0.85, 95% CI = 0.82–0.88; as well as allele comparison model (T vs. C): OR = 0.85, 95% CI = 0.81–0.89] and bladder cancer [dominant (CT+TT vs. CC): OR = 0.83, 95% CI = 0.70–0.99; and allele comparison model (T vs. C): OR = 0.85, 95% CI = 0.75–0.98]. In the subgroup analysis by ethnicity and control source, there was a statistically significant association found in the studies of Asians and Caucasians in the hospital-based and population-based studies under all genetic models.

**Table 2:** Meta-analysis of the association between rs402710 and rs401681 polymorphisms and cancer risk

Variables	No. of individuals	Homozygous			Heterozygous			Recessive			Dominant			Allele		
		TT vs. CC			CT vs. CC			TT vs. CT+CC			CT+TT vs. CC			T vs. C		
		OR (95% CI)	Phet	I^2^ (%)	OR (95% CI)	Phet	I^2^ (%)	OR (95% CI)	Phet	I^2^ (%)	OR (95% CI)	Phet	I^2^ (%)	OR (95% CI)	Phet	I^2^ (%)
**rs402710 polymorphism**																
All	30770/34089	**0.77 (0.70–0.84)**	0.006	46.8	**0.89 (0.86–0.92)**	0.121	26.0	**0.81 (0.74–0.88)**	0.021	40.6	**0.87 (0.83–0.91)**	0.032	37.9	0.88 (0.84–0.92)	< 0.001	65.8
Asian	16851/20254	**0.78 (0.69–0.88)**	0.002	53.6	**0.90 (0.86–0.94)**	0.070	34.0	**0.82 (0.73–0.91)**	0.008	48.5	**0.88 (0.83–0.94)**	0.017	44.6	0.90 (0.85–0.94)	0.002	54.3
African	1980/1911	0.87 (0.56–1.36)	/	/	0.89 (0.68–1.16)	/	/	0.92 (0.60–1.41)	/	/	0.89 (0.69–1.14)	/	/	0.98 (0.89–1.07)	0.435	0.0
Caucasian	11939/11924	**0.73 (0.66–0.80)**	0.634	0.0	**0.86 (0.82–0.92)**	0.661	0.0	**0.78** **(0.72–0.86)**	0.590	0.0	**0.84 (0.79–0.88)**	0.681	0.0	**0.76 (0.66–0.87)**	< 0.001	82.8
Lung	24619/27710	**0.73 (0.67–0.79)**	0.205	21.8	**0.88 (0.85–0.91)**	0.272	15.8	**0.77 (0.71–0.84)**	0.104	32.3	**0.85 (0.82–0.88)**	0.437	1.3	**0.85 (0.81–0.89)**	0.001	58.0
Bladder	974/1055	0.75 (0.55–1.01)	0.347	0.0	0.85 (0.71–1.03)	0.651	0.0	0.80 (0.60–1.08)	0.409	0.0	**0.83 (0.70–0.99)**	0.471	0.0	**0.85 (0.75–0.98)**	0.359	0.0
Others	5177/5324	0.98 (0.74–1.30)	0.009	67.7	0.97 (0.88–1.07)	0.096	46.5	0.97 (0.78–1.20)	0.065	51.8	0.99 (0.84–1.17)	0.011	66.5	0.99 |(0.89–1.10)	0.004	68.6
HB	10754/11408	**0.81 (0.69–0.95)**	0.002	60.1	**0.91 |(0.86–0.97)**	0.185	25.0	**0.84 (0.73–0.97)**	0.016	50.2	**0.90 (0.82–0.97)**	0.017	50.0	**0.90 (0.84–0.96)**	< 0.001	64.6
PB	17357/19837	**0.73 (0.67–0.80)**	0.404	3.7	**0.87 (0.83–0.91)**	0.216	25.6	**0.78 (0.70–0.87)**	0.214	25.8	**0.84 (0.80–0.88)**	0.465	0.0	**0.86 (0.80–0.92)**	< 0.001	71.6
Mixed	2659/2844	**0.74 (0.61–0.89)**	/	/	0.94 (0.84–1.05)	/	/	**0.76** **(0.63–0.91)**	/	/	**0.90 (0.81–0.99)**	/	/	**0.89 (0.82–0.96)**	/	/
**rs401681 polymorphism**																
All	67849/328226	**0.87 (0.76–0.98)**	< 0.001	79.2	0.95 (0.88–1.01)	< 0.001	64.7	**0.90** **(0.81–0.99)**	< 0.001	73.0	0.93 (0.87–1.01)	< 0.001	74.2	**0.93 (0.89–0.97)**	< 0.001	88.8
Asian	19070/21794	**0.76 (0.66–0.87)**	< 0.001	58.9	**0.89 (0.83–0.96)**	0.018	42.9	**0.80** **(0.71–0.92)**	< 0.001	57.5	**0.87 (0.81–0.93)**	0.004	49.7	**0.88 (0.84–0.93)**	0.001	55.1
Caucasian	43266/300853	1.11 (0.90–1.37)	< 0.001	86.3	1.05 (0.92–1.20)	< 0.001	77.6	1.08 (0.93–1.24)	< 0.001	78.4	1.07 (0.92–1.24)	< 0.001	84.4	0.96 (0.90–1.02)	< 0.001	93.3
African	1981/1937	0.71 (0.49–1.03)	/	/	0.88 (0.67–1.16)	/	/	0.77 (0.56–1.07)	/	/	0.83 (0.64–1.08)	/	/	0.95 (0.80–1.13)	0.081	67.1
Lung	24371/55976	**0.73 (0.66–081)**	0.220	22.4	**0.86 (0.81–0.92)**	0.316	12.0	**0.78 (0.70–0.88)**	0.072	36.5	**0.84 (0.80–0.88)**	0.600	0.0	**0.86 (0.84–0.89)**	0.292	13.0
Melanoma	6194/45903	**1.47(1.23–1.75)**	0.291	19.0	**1.24 (1.09–1.41)**	0.976	0.0	**1.29 \** **(1.09–1.53)**	0.196	38.6	**1.31 (1.16–1.47)**	0.747	0.0	**1.19 (1.14–1.24)**	0.451	0.0
Pancreatic	7206/9927	**1.41 (1.24–1.60)**	0.576	0.0	**1.17 (1.06–1.29)**	0.688	0.0	**1.27** **(1.12–1.45)**	0.299	17.2	**1.24 (1.13–1.36)**	0.898	0.0	**1.19 (1.14–1.24)**	0.978	0.0
Bladder	5663/37411	**0.70 (0.56–0.87)**	0.491	0.0	0.89 (0.77–1.03)	0.990	0.0	**0.74 (0.61–0.91)**	0.445	0.0	**0.85 (0.74–0.97)**	0.949	0.0	**0.84 (0.81–0.88)**	0.871	0.0
Esophageal	1357/1524	0.82 (0.64–1.05)	0.395	0.0	0.83 (0.66–1.05)	0.135	55.2	0.90 (0.71–1.14)	0.693	0.0	0.83 (0.66–1.04)	0.121	58.4	0.88 (0.76–1.02)	0.186	42.8
SCC	1369/36633	0.82 (0.53–1.26)	/	/	1.26 (0.92–1.72)	/	/	0.71 (0.48–1.04)	/	/	1.13 (0.84–1.53)	/	/	0.93 (0.86–1.01)	0.733	0.0
BCC	6316/68321	0.87 (0.60–1.27)	/	/	**0.71 (0.53–0.96)**	/	/	1.05 (0.75–1.48)	/	/	**0.76 (0.57–1.00)**	/	/	**0.82 (0.79–0.85)**	0.692	0.0
Others	13521/70523	0.86 (0.57–1.30)	0.007	79.6	1.03 (0.75–1.41)	0.002	84.6	0.88 (0.59–1.30)	0.006	80.2	1.01 (0.74–1.38)	0.001	85.9	**0.92 (0.85–1.00)**	< 0.001	77.1
HB	11849/12401	**0.78 (0.66–0.94)**	< 0.001	63.6	0.92 (0.84–1.01)	0.016	48.5	**0.81 (0.70–0.95)**	0.004	55.4	**0.90** **(0.82–0.99)**	0.002	58.3	**0.90 (0.84–0.97)**	< 0.001	66.9
PB	19325/90701	0.94 (0.79–1.12)	< 0.001	84.3	0.96 (0.87–1.06)	< 0.001	73.5	0.96 (0.84–1.10)	< 0.001	78.8	0.93 (0.87–1.01)	< 0.001	81.0	0.95 (0.88–1.02)	< 0.001	83.5

HB, hospital based; PB, population based; SCC, squamous cell carcinoma; BCC, basal cell carcinoma

### Meta-analysis results of rs401681 polymorphism

**Figure 3:**
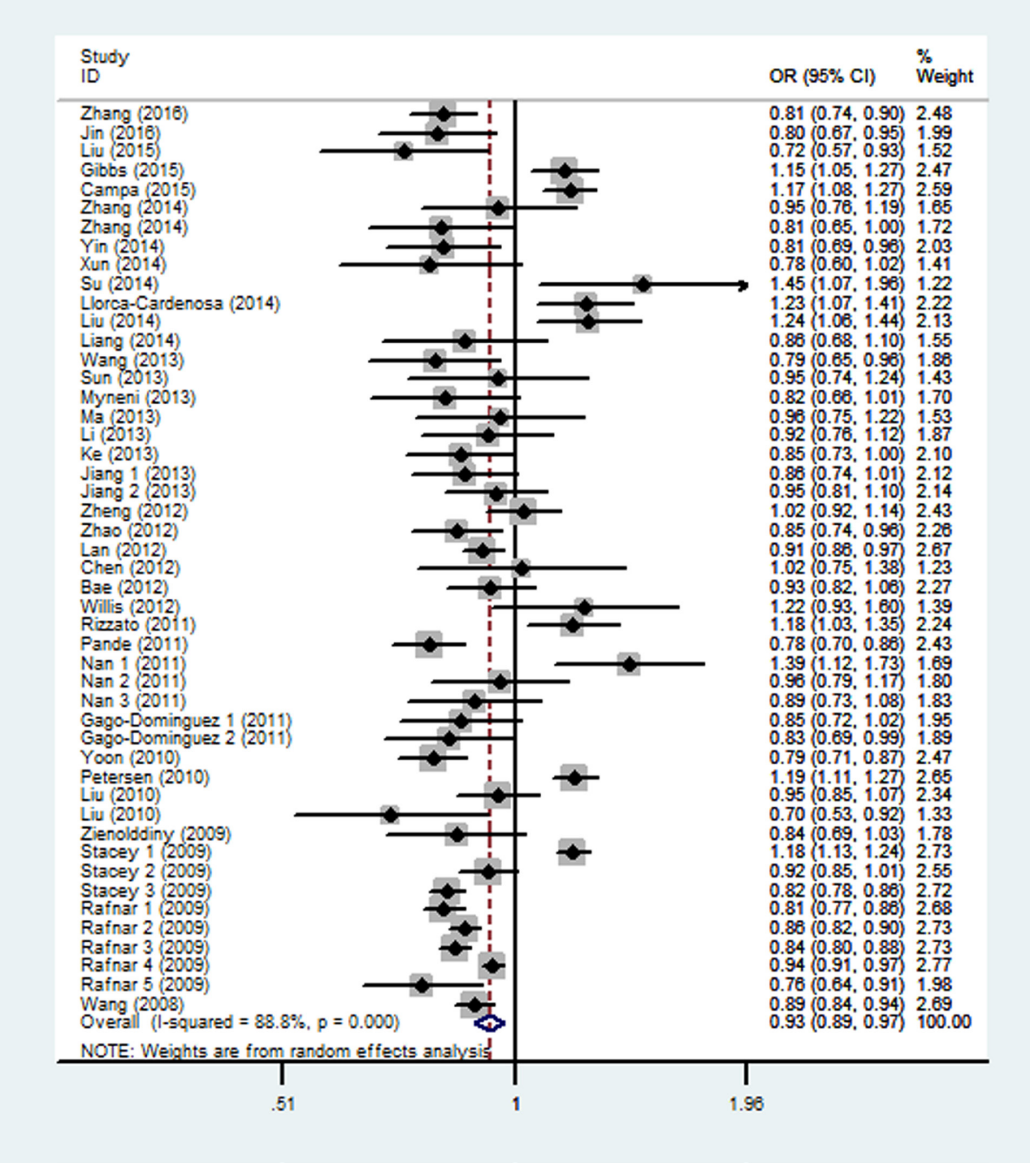
Forest plot of the association between *CLPTM1L* rs401681 polymorphism and overall cancer risk under the allele contrast model

As shown in Table 2 and Figure 3, similar to the rs402710 polymorphism, a significant association between the rs401681 polymorphism and overall cancer risk was also observed [homozygous (TT vs. CC): OR = 0.87, 95% CI = 0.76–0.98; recessive (TT vs. CT+CC): OR = 0.90, 95% CI = 0.81–0.99; and allele comparison model (T vs. C): OR = 0.93, 95% CI = 0.89–0.97]. In the stratification analysis by cancer type, a decreased association was identified with lung cancer [homozygous (TT vs. CC): OR = 0.73, 95% CI = 0.66–0.81; heterozygous (CT vs. CC): OR = 0.86, 95% CI = 0.81–0.92; recessive (TT vs. CT+CC): OR = 0.78, 95% CI = 0.70–0.88; and dominant (CT+TT vs. CC): OR = 0.84, 95% CI = 0.80–0.88; as well as allele comparison model (T vs. C): OR = 0.86, 95% CI = 0.84–0.89], bladder cancer and basal cell carcinoma. On the contrary, there was an increased risk for melanoma [homozygous (TT vs. CC): OR = 1.47, 95% CI = 1.23–1.75; heterozygous (CT vs. CC): OR = 1.24, 95% CI = 1.09–1.41; recessive (TT vs. CT+CC): OR = 1.29, 95% CI = 1.09–1.53; and dominant (CT+TT vs. CC): OR = 1.31, 95% CI = 1.16–1.47; as well as allele comparison model (T vs. C): OR = 1.19, 95% CI = 1.14–1.24] and pancreatic cancer [homozygous (TT vs. CC): OR = 1.41, 95% CI = 1.24–1.60; heterozygous (CT vs. CC): OR = 1.17, 95% CI = 1.06–1.29; recessive (TT vs. CT+CC): OR = 1.27, 95% CI = 1.12–1.45; and dominant (CT+TT vs. CC): OR = 1.24, 95% CI = 1.13–1.36; as well as allele comparison model (T vs. C): OR = 1.19, 95% CI = 1.14–1.24]. When the analysis was stratified by ethnicity, there was a statistically significant association among Asians, but not in Caucasians or Africans. When the analysis was stratified by control source, there was a statistically significant association in the hospital-based studies, but not in population-based studies.

### Heterogeneity and sensitivity analyses

Substantial heterogeneities were observed among all studies of the rs402710 polymorphism and cancer risk (homozygous: *p* = 0.006; recessive: *p* = 0.021; and dominant: *p* = 0.032; as well as allele comparison model: *p* < 0.001), except for the heterozygous model (*p* = 0.121). As to the rs401681 polymorphism, we also observed considerable heterogeneities (homozygous: *p* < 0.001; heterozygous: *p* < 0.001; recessive: *p* < 0.001; and dominant: *p* < 0.001; as well as allele comparison model: *p* < 0.001). So, the random-effects model was applied to generate crude ORs and 95% CIs. In addition, the leave-one-out sensitivity analysis indicated that no single study altered the corresponding pooled ORs and 95% CIs.

### Publication bias

The shape of our funnel plot did not reveal any evidence of obvious asymmetry for the rs402710 polymorphism (homozygous: *p* = 0.738; heterozygous: *p* = 0.291; recessive: *p* = 0.954; and dominant: *p* = 0.318; as well as allele comparison model: *p* = 0.946) and rs401681 polymorphism (homozygous: *p* = 0.264; heterozygous: *p* = 0.451; recessive: *p* = 0.114; and dominant: *p* = 0.564; as well as allele comparison model: *p* = 0.967).

## DISCUSSION

The *CLPTM1L* gene encodes the CLPTM1-like protein which is highly expressed in cisplatin-resistant ovarian tumor cell lines and is associated with cisplatin-induced apoptosis [8]. This gene has also been demonstrated to be overexpressed in many other cancers [58–61]. Blockade of CLPTM1-like protein has been shown to inhibit K-Ras-induced lung tumorigenesis [61]. *CLPTM1L*, near the *TERT* gene, is located at chromosome 5p15.33 which is widely identified as a susceptibility region associated with several types of cancer [62]. Some common genetic variants of *TERT-CLPTM1L* are hypothesized to have an important role in initiation and development of many cancers, including lung, bladder, pancreatic, thyroid, and breast cancer.

To the best of our knowledge, the meta-analysis in this study is the largest to study the associations between *CLPTM1L* rs402710 (C>T) and rs401681 (C>T) polymorphisms and overall cancer risk. In this meta-analysis, based on the database of publications in respect of the rs402710 polymorphism and the rs401681 polymorphism, among which there are 26 articles with 28 studies including 30,770 cases and 34,089 controls for rs402710 polymorphism and 38 articles with 48 studies including 67,849 cases and 328,226 controls for rs401681 polymorphism respectively, we observed both rs402710 and rs401681 polymorphisms were significantly associated with a decreased overall cancer risk. In the stratification analysis, as to the rs402710 polymorphism, a statistically significant association was also identified for lung and bladder cancer. Likewise, the rs401681 polymorphism was significantly associated with a decreased risk for lung cancer, bladder cancer and basal cell carcinoma. Interestingly, an increased risk of melanoma and pancreatic cancer was found for the rs401681 polymorphism.

Up until now, there had been only one meta-analysis (in 2013) focusing on rs402710 and rs401681 polymorphisms and overall cancer risk [62]; which only enrolled seven studies with 4,667 cancer patients and 4,990 controls for the rs402710 polymorphism and eight studies with 6,867 cancer patients and 7,746 controls for the rs401681 polymorphism. The said study found that rs402710 (A>G) and rs401681 (A>G) polymorphisms were associated with an increased cancer risk. In other words, rs402710 (C>T) and rs401681 (C>T) polymorphisms were significantly associated with a decreased overall cancer risk. In the stratification analysis by cancer type, Li et al. [62] included a few studies and did further stratification analysis only for lung and bladder cancer. Such associations were also observed with lung cancer from the meta-analyses before 2014 [63–65]. Since then, at least 8 studies for the rs402710 polymorphism and 13 studies for the rs401681 polymorphism have been published investigating the association with overall cancer risk, most of which focused on lung cancer. As the largest study with the strongest statistical power so far, the current meta-analysis also observed an association between the rs401681 polymorphism and an increased risk for melanoma and pancreatic cancer, which had been found in other meta-analyses [66, 67]. This observation suggested that the rs401681 polymorphism could play different roles in different types of cancer.

Several limitations in our meta-analysis should be acknowledged. First, we only included the studies published in English, which may have missed publications in other languages. Second, in the stratification analysis by cancer type and ethnicity, the sample sizes are relatively small and may be insufficient, which may have diminished the statistical power. Third, due to the lack of raw information such as age, sex, body mass index, smoking habits, and alcohol consumption, this meta-analysis was conducted on the basis of unadjusted estimates. Therefore, larger and well-designed studies with different types of cancer and multiple ethnic with sufficient raw information are warranted to validate the findings of this study.

In conclusion, the meta-analysis in this study demonstrated that *CLPTM1L* gene rs402710 and rs401681 polymorphisms were associated with a decreased risk of various types of cancer, especially for lung cancer among Asians.

## MATERIALS AND METHODS

### Search strategy

PubMed and EMBASE electronic databases were screened for publications investigating the association between *CLPTM1L* polymorphisms and overall cancer risk published prior to May 1, 2017. The following search terms were used: “CLPTM1L”, “5p15”, “polymorphism”, “variant”, “cancer”, “tumor” and “carcinoma”. Additional relevant studies were also retrieved manually from all the relevant publications, including the eligible original publications and reviews. Only the latest or the largest study would be included in the final meta-analysis.

### Inclusion and exclusion criteria

All studies included in the current meta-analysis met the following criteria: (1) evaluating the association between *CLPTM1L* rs402710 and rs401681 polymorphisms and the risk of all types of cancer; (2) case-control or cohort studies; (3) published in English; (4) including sufficient data for genotype frequencies; and (5) providing sufficient data to calculate ORs and 95% CIs. Exclusion criteria include: (1) insufficient raw data; (2) meta-analyses, reviews and comments; (3) duplicates of previous publications; and (4) case-only studies. Studies that were departure from HWE in controls were also excluded, unless further evidence indicated that another polymorphism was in agreement with HWE.

### Data extraction

Two investigators extracted the following information from all the eligible studies independently: surname of the first author, publication year, country of origin, ethnicity, cancer type, control source, genotyping method, number of cases and controls, and genotype frequencies of cases and controls. Different ethnicities were stratified into Asian, African, and Caucasian. The source of controls was categorized as hospital-based and population-based studies. Any disagreements were discussed and resolved until the consensus was reached.

### Statistical analysis

The strength of association between the *CLPTM1L* rs402710 and rs401681 polymorphisms and the risk of all types of cancer were assessed by the calculation of crude ORs and 95% CIs. Stratification analyses were further performed according to ethnicity, cancer type and control source. The pooled ORs and 95% CIs were estimated for rs402710 polymorphism under the homozygous (TT vs. CC), heterozygous (CT vs. CC), recessive (TT vs. CT+CC), and dominant (CT+TT vs. CC), as well as allele comparison model (T vs. C) and so was rs401681 polymorphism under the homozygous (TT vs. CC), heterozygous (CT vs. CC), recessive (TT vs. CT+CC), and dominant (CT+TT vs. CC), as well as allele comparison model (T vs. C).

The Chi square-based Q-test was applied to evaluate between-study heterogeneity. If the studies were found to have no heterogeneity (*p* > 0.10), the fixed-effects model (the Mantel-Haensze method) was used [68]. Otherwise, the random-effects model (the DerSimonian and Laird method) was adopted [69]. Furthermore, heterogeneity was also identified with the I^2^ test [70]. The higher values suggest the greater degree of heterogeneity. Thus the publication bias was quantified by Begg's funnel plot and Egger's linear regression test [71].

All the statistical analyses were conducted using STATA software (version 12.0, STATA Corporation, College Station, TX) and were two-sided, with *P* values less than 0.05 as statistically significant.
